# Evolution of the Soil Bacterial Community as a Function of Crop Management: A Metagenomic Study in Orange Tree (*Citrus sinensis*) Plantations

**DOI:** 10.3390/plants14121781

**Published:** 2025-06-11

**Authors:** Carlos Giménez-Valero, Alejandro Andy Maciá-Vázquez, Dámaris Núñez-Gómez, Agustín Conesa, Vicente Lidón, Pablo Melgarejo

**Affiliations:** Plant Production and Microbiology Department, Escuela Politécnica Superior de Orihuela (EPSO-UMH), Miguel Hernández University, Ctra. Beniel km 3.2, 03312 Orihuela, Spain

**Keywords:** metagenomics, weed control netting, bacterial diversity, bacterial community, semi-arid agroecosystems, sustainable agriculture

## Abstract

Soil management significantly influences the structure and diversity of soil bacterial communities, affecting biodiversity and ecosystem functions. In semi-arid regions, water efficiency strategies like anti-weed netting are implemented, but their impact on soil microbial communities remains underexplored. This study evaluates the temporal evolution of soil bacterial communities in orange tree (*Citrus sinensis* (L.) Osbeck) plantations under two conditions: with and without anti-weed netting. Soil samples were collected at three time points over a period of 18 months since the establishment of the crop and analyzed using high-throughput 16S rRNA sequencing, assessing alpha and beta diversity, taxonomic composition, and functional pathways via KEGG analysis. The results indicate that weed control netting contributes to stabilizing bacterial diversity over time and increases the relative abundance of dominant phyla such as Planctomycetota, Proteobacteria, Bacteroidota, and Acidobacteriota. Functional predictions revealed significant differences in metabolic pathways, including those associated with nitrogen fixation and organic matter degradation. These findings suggest that anti-weed netting not only influences the taxonomic composition of soil bacterial communities but also modulates their functional potential, with implications for sustainable agriculture in semi-arid environments. This study provides new insights into the interaction between soil management and soil bacterial communities, offering valuable information for optimizing agricultural practices and soil conservation strategies.

## 1. Introduction

Soil is a complex and dynamic ecosystem whose bacterial community plays a key role in fertility, nutrient recycling, and resilience to adverse environmental factors [1]. Soil bacterial biodiversity is influenced by multiple biotic and abiotic factors, including agricultural management practices. It has been documented that soil bacteria actively participate in processes such as mineralization of organic matter, biological nitrogen fixation, and degradation of xenobiotic compounds, which are essential functions for the stability of agroecosystems [1,2]. However, agricultural intensification and modification of soil conditions through different management practices can drastically alter the structure and functionality of the microbial community, affecting its ability to maintain key biogeochemical cycles and its resilience to environmental perturbations [3].

One of the widely used approaches in agriculture to optimize soil management is the use of anti-weed netting, a plastic mulch system designed to minimize weed competition, reduce evaporation, and improve water efficiency in perennial crops such as sweet orange. However, the effects that this practice may have on microbial biodiversity and soil functionality over time are still unknown. Previous studies have shown that the use of plastic mulches can influence soil physicochemical conditions, affecting water, temperature, and nutrient availability, which, in turn, can modify the composition and activity of soil bacterial communities [4]. While some authors have reported that these covers can reduce microbial diversity by generating anaerobic microenvironments and altering oxygen availability [5], others have suggested that they favor the proliferation of communities specialized in the degradation of organic matter and the stabilization of soil aggregates [6].

The southeast of Spain is one of the main agricultural regions of the country, characterized by a semi-arid climate with low rainfall and high temperatures [7]. Citrus farming is a very important agricultural sector in this region, with the Valencian Community, Murcia, and Andalusia as the main producing areas. Spain is the main citrus producer in the European Union and the sixth-largest producer in the world, with a production of approximately 2.75 million tons of oranges in 2023. In addition, the southeastern Spanish area reached a historic record in fruit and vegetable exports in 2024, exceeding EUR 3.5 billion, with citrus fruits standing out as a key product. Efficient water management and the implementation of sustainable agricultural practices are essential for maintaining the productivity and quality of citrus crops in this region. In this context, the present study analyzes the impact of soil cover using anti-weed nets on soil bacterial communities in sweet orange crops, evaluating its effect on microbial diversity and stability. Semi-arid regions have soils that are highly vulnerable to erosion and desertification processes, which makes it necessary to manage agriculture that seeks not only to maximize yield but also to conserve microbial biodiversity and soil functionality [8]. The bacterial community in soil contributes significantly to agricultural resilience in water-limited environments by enhancing soil stabilization and water retention [9]. However, information on how weed control nets influence soil metagenomic evolution remains limited, especially in perennial crops such as orange trees. This study not only contributes to understanding how this agricultural practice affects microbial biodiversity, but also provides a detailed temporal assessment of its evolution, using high-throughput metagenomic approaches to analyze both the taxonomic composition and functionality of soil bacterial communities. It is hypothesized that soil coverage with anti-weed netting induces significant changes in the diversity and functional potential of soil bacterial communities, thereby modulating their temporal dynamics.

When performing mechanized soil preparation work for a new orange grove, and then installing weed control netting, partially covering the soil under the trees, one might initially think that the local conditions produced by the netting may not be ideal for the growth and development of bacterial communities. Based on this premise, the study aimed to (i) evaluate the temporal evolution of the soil bacterial community in orange tree (*Citrus sinensis*) plantations under contrasting management conditions (with and without anti-weed netting), (ii) analyze alpha and beta diversity over an 18-month period, (iii) assess the taxonomic composition and changes in the relative abundance of dominant bacterial phyla, and (iv) investigate the functional potential of the bacterial community through the analysis of KEGG metabolic pathways in relation to soil physicochemical conditions.

Multiple important reasons compel agriculture in southeastern Spain to seek alternatives and incorporate new cultivation techniques, ideally integrated into three main areas: improving yields, increasing profitability, and respecting the environment. Among the factors involved are limited water availability and the need to incorporate innovations in agricultural practices and strategies, which can directly or indirectly induce modifications in soil properties, whose effect on the structure and functionality of soil bacterial communities remains largely uncharacterized, among other aspects. However, in general terms, this study suggests that installing the anti-weed mesh does not produce such a marked negative impact on bacterial communities, favoring the growth and development of bacteria under the mesh, even under better conditions than if it were not installed. Therefore, farmers can benefit from all the positive aspects that its installation provides, knowing that the soil will retain the microbial communities that are so important, both for cultivation and for natural soil conservation.

## 2. Materials and Methods

### 2.1. Experimental Design and Soil Samples

The soil samples for this study were collected from an experimental agricultural plot located at the High Polytechnic School of Orihuela (EPSO) of Miguel Hernandez University (UMH) (Spain).

To evaluate the evolution of soil bacteria in a *Citrus sinensis* L. Osbeck crop, an experimental design was established based on two study variables: sampling time and the presence or absence of anti-weed mesh installed under the crop.

For the time factor, three sampling points were defined: Time 0 (S0), prior to planting; Time 1 (T1), corresponding to 9 months after planting; and Time 2 (T2), corresponding to 18 months after planting. The impact of the anti-weed netting was assessed through two management treatments: cultivation without netting (S1), representing traditional management without soil cover, and cultivation with anti-weed netting (S2), designed to suppress weed growth, reduce runoff, retain moisture, and regulate soil temperature (Table 1).

To collect soil samples from group S2, the weed control mesh had to be punctured twice: once during the first 9 months and again during the following 9 months. The trees are planted in the linear center of the weed control mesh, and the mesh is secured and anchored to the soil at the buried lateral ends along the entire length of the mesh. The installed weed control mesh is 4 m wide, woven with polypropylene bands, and has a weight of 150 g/m^2^. It offers high resistance to tearing and puncture. It is water-permeable and UV-treated, allowing it to remain outdoors without the need for coating.

Once the orange trees were planted in the experimental plot and all the necessary equipment was installed, we noted Time 0, the moment in which the orange trees were planted. During that same week, the first 5 samples belonging to the control group (S0-1, S0-2, S0-3, S0-4 and S0-5) were collected, and together with these samples, a physical–chemical analysis and a subsequent agronomic assessment of the cultivated soil were carried out. All soil samples were obtained using a drilling auger at a standard depth of 45 cm from the soil surface in an area close to the tree trunks, ensuring the representativeness of the rhizospheric microbiome. Following the experimental design and after 9 months of cultivation from Time 0, a total of 10 samples were collected for the second part of the study. The samples were collected in two groups of 5. The first group consisted of 5 samples (S1-T1-1, S1-T1-2, S1-T1-3, S1-T1-4, and S1-T1-5), representing traditional cultivation without weed control netting (S1). The second group also consisted of 5 samples (S2-T1-1, S2-T1-2, S2-T1-3, S2-T1-4, and S2-T1-5), belonging to the cultivation block with weed control netting covering the soil (S2). The T1 designation corresponds to 9 months. Finally, for the last phase of the study and after 18 months from Time 0, another 10 samples were collected for the same previous groups, 5 samples for cultivation without netting (S1-T2-1, S1-T2-2, S1-T2-3, S1-T2-4 and S1-T2-5) and another 5 for cultivation with anti-weed netting (S2-T2-1, S2-T2-2, S2-T2-3, S2-T2-4 and S2-T2-5). It must be noted that the designation T2 corresponds to 18 months.

To ensure the representativeness of each sample, a total of 25 samples of 400–500 g each were collected per study block (S0, S1, and S2). Subsequently, the samples belonging to each block were mixed carefully, and finally, five independent aliquots were extracted for each of the variables studied (*n* = 25) to perform the pertinent analytical determinations. In treatments with weed control mesh (S2-T1 and S2-T2), incisions were made in the mesh for soil collection, ensuring that the analyzed bacterial community corresponded to the soil profile in direct contact with the crop root system. The selection of trees and sampling points followed a stratified random design, considering the location of the irrigation drippers and equitably distributing the extraction points in relation to the cardinal orientation. The soil samples were stored in an ultra-freezer at −80 °C until laboratory analysis. In the treatments with an anti-weed mesh (S2-T1 and S2-T2), incisions were made in the mesh to collect soil samples, ensuring that the bacterial community analyzed corresponded to the soil profile in direct contact with the root system of the crop.

During the sampling period, all trees were in good phytosanitary condition, with no obvious symptoms of pests or diseases, which allowed us to rule out possible adverse effects on the soil bacterial community due to plant health problems. In addition, the soil samples were agronomically characterized using physical–chemical analysis by an external laboratory certified and approved in Spain.

This experimental scheme allows us to evaluate (i) the temporal evolution of the soil bacterial community under sweet orange cultivation and (ii) the impact of the anti-weed mesh on the soil microbial composition and dynamics.

### 2.2. Agronomic Characterization of Soil Samples

The results obtained indicated that the soil has a clayey-loam texture, according to the USDA classification [10], with a composition of 34.15% sand, 33.35% clay, and 32.50% silt, which defines it as a medium-textured soil. The soil was characterized as being slightly alkaline, with a pH of 8.00, determined according to ISO 10390 [11]. The analysis was performed by preparing a 1:5 soil-to-deionized water suspension, which was agitated, allowed to equilibrate, and then measured using a calibrated benchtop pH meter (HI 3220 + mV/°C, Hanna^®^ Instruments, Eibar, Gipuzkoa, Spain) equipped with a glass electrode. The electrical conductivity was 2.36 mS/cm, measured in a 1:2 soil-to-water extract following UNE-EN 13038 [12]. Conductivity was assessed using a calibrated conductivity meter with automatic temperature compensation, ensuring accurate measurement of the soil’s soluble salt content.

The apparent bulk density was 1.42 g/cm^3^, while the carbon-to-nitrogen (C/N) ratio was 11.07, providing a reference for the mineralization potential of organic matter in the soil. From a chemical perspective, the soil showed a very high content of sulphates (1670.40 mg/kg) and chlorides (184.60 mg/kg), as well as high levels of sodium (159.85 mg/kg) and calcium (576.00 mg/kg). All measurements were performed in a 1:2 soil-to-deionized water extract, prepared by mixing 10 g of air-dried, sieved soil with 20 mL of deionized water, followed by agitation and filtration to remove particulates. Separation of anions was performed using a DIONEX ICS-1000 ion chromatography system (Thermo Fisher Scientific, Waltham, MA, USA) equipped with a conductivity detector and an IonPac AS11-HC analytical column (4 × 250 mm) with a matching AG11-HC guard column (4 × 50 mm), using isocratic elution with 30 mM NaOH at a flow rate of 1.0 mL/min at 30 °C. Eluent suppression was achieved using an AMMS-ICE suppressor. The injection volume was 25 µL. The data were collected and processed using Chromeleon software (version 7.3, Thermo Fisher Scientific, Massachusetts, USA). This methodology follows the procedures for environmental and agronomic analysis [13]. This analytical approach allows for precise quantification of soluble ionic species, and the concentrations obtained indicate that the soil can be classified as saline, a condition that may influence nutrient availability and crop physiological responses.

Additionally, the soil had a very high total limestone content of 56.90%, determined by volumetric analysis using a Bernard calcimeter, based on the reaction of carbonates with hydrochloric acid and the measurement of the volume of CO_2_ released [14,15]. Organic matter content was quantified using the loss-on-ignition method, which involved drying the sample at 105 °C and combusting it at 550 °C in a muffle furnace to estimate the organic carbon content by weight loss [14,15]. The result, a low value of 1.43%, suggests limited biological activity and a reduced capacity to retain moisture and nutrients compared to soils with higher organic matter levels. All analyses were conducted on air-dried, sieved samples (<2 mm), following standard soil science protocols.

All analyses and determinations performed to characterize this soil were carried out by a laboratory accredited by the Spanish National Accreditation Entity (ENAC). For environmental testing, the laboratory complies with the UNE-EN ISO/IEC 17025 standard [16].

### 2.3. rRNA Sequencing

To characterize the composition and structure of the microbial communities present in the soil samples, amplification and sequencing of the V3-V4 hypervariable regions of the 16S rRNA gene were carried out [17].

DNA amplification was performed using polymerase chain reaction (PCR) with 25 cycles, ensuring adequate amplification of the target regions [18].

Subsequently, the generated libraries were sequenced on an Illumina MiSeq platform, using a 300 × 2 bp paired-end sequencing protocol, ensuring adequate coverage for metagenomic characterization of the soil microbiome.

### 2.4. Bioinformatics Procedures and Analysis

The data obtained were analyzed using QIIME2 [19]. Data quality was assessed and processed with DADA2 [20] by removing adapters, filtering low quality, removing noise, and assembling forward and reverse reads for amplicon reconstruction. Taxonomic classification was performed using a Bayesian classifier [21] trained with the Silva v.138 database (99% OTUs) [22].

Alpha diversity was assessed using richness (number of observed OTUs) and evenness (Pielou index) metrics, compared with a generalized linear model (GLM) using the MASS v.7.3-54 [23] and glmmTMB v.1.1.8 packages in R. Beta diversity was determined using unweighted Unifrac, weighted Unifrac, Jaccard, and Bray–Curtis distances with PERMANOVA, ANOSIM, and PERMDISP tests to assess the significance of differences between groups [24]. Beta diversity distance matrices were used to calculate principal coordinate analysis (PCoA) and to create ordination plots using the R software package version 4.2.0.

**Figure 1:**
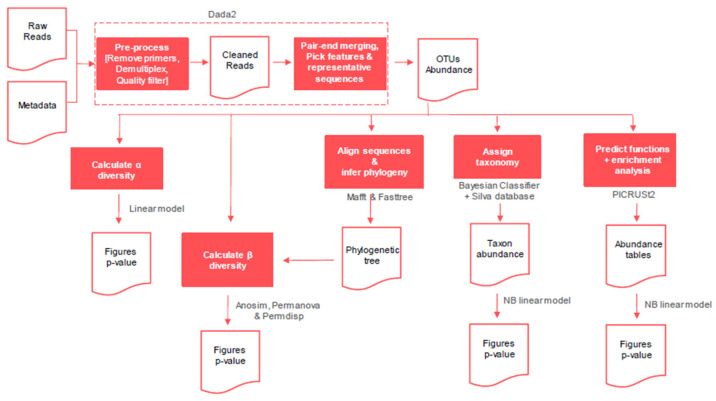
Workflow followed for the data analysis.

Phylogenetic relationships were established using Mafft [25] and FastTree [26]. The functionality of the microbial community was inferred with PICRUSt2 [27], estimating gene abundance and metabolic pathways based on the KEGG database [28]. Finally, differential abundance analysis was carried out with negative binomial distribution generalized linear models (NB-GLM) using the MASS and NBZIMM v.1.0 [29] packages in R. Figure 1 presents the workflow followed for the data analysis.

## 3. Results

### 3.1. Soil Bacterial Community Without Anti-Weed Mesh (S0, S1-T1, S1-T2)

#### 3.1.1. Alpha Diversity: Variations in Soil Bacterial Community: Richness and Evenness

The alpha diversity of bacterial communities in soil without an anti-weed mesh was quantified using the number of operational taxonomic units (OTUs) observed, while the evenness in the distribution of communities was assessed using the Pielou evenness index. This analysis allows us to determine the metagenomic evolution of the soil over the 18 months of the study.

The results showed significant differences in OTU richness between samples S0 and S1-T1 (*p* = 0.02), according to the negative binomial distribution model, as well as differences in evenness (*p* = 0.02), according to the beta regression model. The OTU richness in S0 was 636.4 (±254.54), significantly reducing in S1-T1 to 388.0 (±234.19) OTUs. However, no significant differences were detected between S0 and S1-T2, whose richness was 575.0 (± 111.02) OTUs, nor in evenness (Figure S1, Material Supplementary).

The Pielou evenness index reflected the lowest evenness in S1-T1 (0.952) compared to S0 (0.956) and S1-T2 (0.954).

#### 3.1.2. Beta Diversity: Changes in Structure and Phylogenetic Differentiation

Beta diversity measures differences in microbial composition between samples by comparing them with each other. To quantify these intersample differences, four distance matrices were calculated: unweighted Unifrac, weighted Unifrac, Bray–Curtis, and Jaccard.

Unweighted Unifrac uses phylogenetic information to compare samples, measuring the phylogenetic distance between sets of taxa in a phylogenetic tree as the fraction of the branch length of the tree that leads to descendants from one environment or the other, but not both. This approach considers the presence/absence of OTUs. Weighted Unifrac is a quantitative version of unweighted Unifrac, which additionally weights the relative abundance of OTUs [30]. Bray–Curtis is a quantitative measure that takes into account both composition and relative abundance, while Jaccard is a qualitative measure based exclusively on the presence/absence of OTUs.

**Figure 2:**
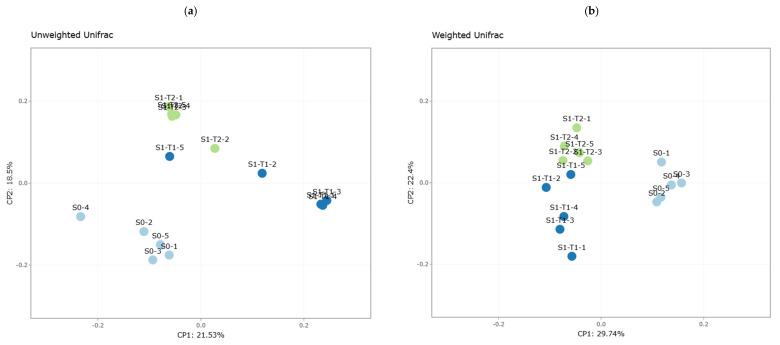

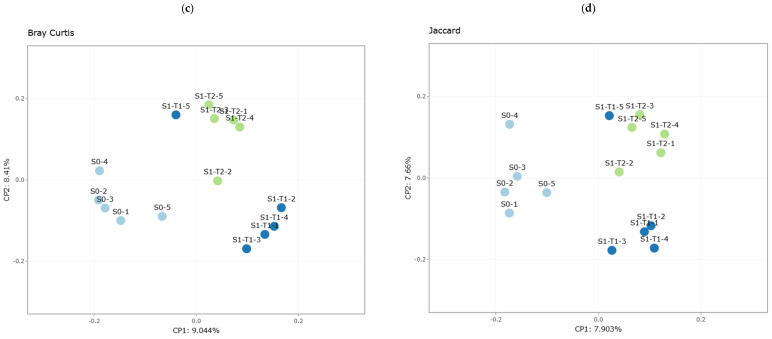
Principal coordinate analysis (PCoA) based on different beta diversity metrics for sample groups S0, S1-T1, and S1-T2. Each sample group is represented by a different color to facilitate interpretation. (**a**) Unweighted Unifrac, (**b**) weighted Unifrac, (**c**) Bray-Curtis, and (**d**) Jaccard. The distance between the points in each graph reflects the differences in microbial composition between the analyzed samples.

The visualization of beta diversity results was performed using principal coordinate analysis (PCoA) (Figure 2). PCoA allows the visualization of similarities or dissimilarities between samples based on a distance matrix. Each sample is represented as a point in a lower-dimensional space, where the distance between points reflects the ecological differences between samples. Each sample group was assigned a distinctive color, facilitating the identification of clusters.

In all the PCoA graphs obtained, a clear separation was observed between the three sample groups S0, S1-T1, and S1-T2 (Figure 2a–d), with a well-defined grouping within each study block as a function of time. In the Unifrac graphs, the samples presented a very close intragroup phylogenetic relationship, except S1-T1, where two samples showed greater phylogenetic dispersion.

#### 3.1.3. Taxonomic Composition and Relative Abundance Patterns

The taxonomic analysis of samples S0, S1-T1, and S1-T2 allowed the identification of a total of 35 phyla, 102 classes, 224 orders, 328 families, 535 genera, and 1014 species in all soil samples analyzed (*n* = 15).

The bacterial dominant phyla in all soil samples, in decreasing order of relative abundance, were Planctomycetota, Proteobacteria, Actinobacteriota, Bacteroidota, Acidobacteriota, Chloroflexi, Gemmatimonadota, Verrucomicrobiota, Crenarchaeota, and Myxococcota. These results are consistent with previous studies [31], in which these phyla represented more than 93% of the readings obtained. In the S1-T1 group, this proportion was reduced to 88.27% (Table S1, Supplementary Material). The difference observed between samples S0 and S1-T2 with respect to S1-T1 is largely attributed to the Firmicutes phylum, whose relative abundance was less than 1.5% in S0 and S1-T2, while in S1-T1, it exceeded 7%. Furthermore, the phyla Methylomirabilota, Firmicutes, Patescibacteria, Nitrospirota, Others, Latescibacterota, Desulfobacterota, Bdellovibrionota, and Sumerlaeota did not exceed 6.11% of total relative abundance in S0 and S1-T2, while in S1-T1, they reached 11.51%.

### 3.2. Soil Bacterial Community with Anti-Weed Mesh (S0, S2-T1, S2-T2)

#### 3.2.1. Alpha Diversity: Temporal Effects on the Bacterial Community

The analysis of alpha diversity in the soil samples with an anti-weed mesh allows us to evaluate the metagenomic evolution from Time 0 to 18 months of the study. The results showed significant differences in terms of evenness between samples S0 and S2-T1 (*p*-value = 0.02), according to the beta regression model applied. The OTU richness was 644.2 (±266.46) for S0, 622.2 (±413.80) for S2-T1, and 720.6 (±214.10) for S2-T2, as shown in Figure S3, in Supplementary Materials. The Pielou evenness index was calculated at 0.957 for S0, 0.952 for S2-T1, and 0.956 for S2-T2. No significant differences were found between S0 and S2-T2 in terms of richness or uniformity. The Pielou index in S2-T1 was the lowest of the three study blocks (0.952).

#### 3.2.2. Beta Diversity: Diversity Between Samples S0, S2-T1, and S2-T2

**Figure 3:**
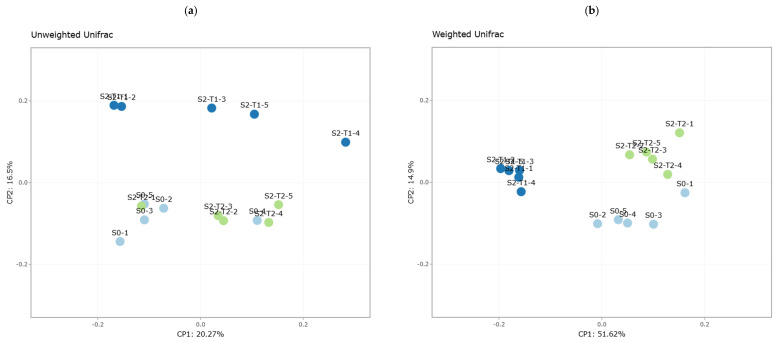

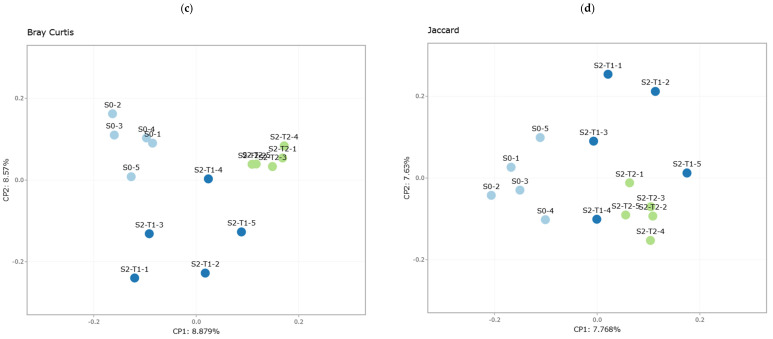
Principal coordinate analysis (PCoA) based on different beta diversity metrics for sample groups S0, S2-T1, and S2-T2. Each group is represented by a different color. (**a**) Unweighted Unifrac, (**b**) weighted Unifrac, (**c**) Bray-Curtis, (**d**) Jaccard. The distance between the points in each graph reflects the differences in microbial composition between the analyzed samples.

The analysis of beta diversity allowed us to assess the differences in bacterial composition between the S0, S2-T1, and S2-T2 sample groups (Figure 3).

In the Unifrac plots, it is evident that the samples present a close phylogenetic relationship within each group. In particular, it is observed that S0 and S2-T2 show greater proximity in terms of taxonomic composition, suggesting similarity in the microbial structure between these conditions. However, in the unweighted Unifrac plot (Figure 3a), samples S2-T1 present greater phylogenetic dispersion. In the weighted Unifrac plot (Figure 3b), a differentiation is also observed in S2-T1, although with similar abundances between the three sample groups.

The Bray–Curtis analysis (Figure 3c) reveals that groups S0 and S2-T2 present a greater similarity in terms of the relative abundance of OTUs, while S2-T1 shows greater dispersion. On the other hand, the Jaccard index (Figure 3d) shows that groups S0 and S2-T2 contain a greater presence of phylogenetically similar taxa.

#### 3.2.3. Taxonomic Profile in S0, S2-T1, and S2-T2: Relative Abundance

In soil samples S0 and S2 (Times 1 and 2), a total of 37 phyla, 105 classes, 241 orders, 355 families, 588 genera, and 1131 species (*n* = 15) were identified. The bacterial dominant phyla in all samples, in decreasing order of relative abundance, were Planctomycetota, Proteobacteria, Actinobacteriota, Bacteroidota, Acidobacteriota, Chloroflexi, Gemmatimonadota, Verrucomicrobiota, Crenarchaeota, and Myxococcota, representing more than 90% of the readings performed, with similar results obtained in other studies [32]. However, in the S2-T1 group, these phyla accounted for 85.52% (Table S2, Supplementary Materials).

## 4. Discussion

### 4.1. Dynamics of Soil Bacterial Communities Without Anti-Weed Mesh (S0, S1-T1, S1-T2)

The observed changes in alpha diversity between S0 and S1-T1 could be attributed to agricultural work prior to the new plantation, such as deep tillage, soil profile thinning, soil aeration, exposure to direct solar radiation, evaporation of soil moisture content, crumbling and breaking of aggregates, as well as soil movement and leveling [33,34]. These factors have been previously described as being responsible for drastic changes in soil microbial composition due to the alteration of soil habitats [35].

The impact of these alterations has been reported in previous studies, where mechanical soil disturbance reduces available organic matter and alters microbial structure, particularly affecting sensitive phyla such as Actinobacteriota and Bacteroidota, while favoring the proliferation of opportunistic taxa such as Firmicutes [36]. These findings are consistent with the observed decrease in OTU richness and altered Pielou evenness in S1-T1 compared to S0 and S1-T2.

Unifrac plots also reflected a close phylogenetic relationship between S0 and S1-T2 taxa, while S1-T1 was markedly differentiated, suggesting a transient restructuring of microbial communities. These results are consistent with the decrease in evenness observed in the Pielou index for S1-T1. Previous studies have reported that beta diversity is highly sensitive to changes in edaphic conditions and soil management, influencing microbial composition through the alteration of ecological habitats [37].

Furthermore, the robustness of the Unifrac and Bray–Curtis metrics to detect changes in microbial diversity has been widely validated in ecological studies, showing a high capacity to identify variations in community composition based on environmental gradients and agricultural practices [30].

Finally, the transient restructuring of the bacterial community observed in S1-T1 and its subsequent tendency for recovery in S1-T2 could be explained by bacterial resilience mechanisms, which have been documented in agricultural systems after soil disturbances [38]. This process suggests that despite initial disturbance, soil bacterial communities can partially recover their structure under stable environmental conditions over time.

The analysis in Table 2 indicates that in six of the twelve main taxa that make up the bacterial communities, the temporal variable had a significant impact on the relative abundance, evidencing fluctuations in the taxonomic composition throughout the study.

One of the most relevant findings is the predominance of the Planctomycetota phylum from Time 0 to Time 2, which differs from previous studies where other phyla were identified as dominant in similar agricultural systems (Figure S2, Supplementary Materials) [39,40]. This observation may be related to the high concentration of sulfate anions (SO_4_^2−^) in the soil studied, since previous research has reported that Planctomycetota tends to proliferate in environments with high sulfate availability [41,42,43].

Additionally, the irrigation water used in the experimental plot comes from the lower section of the Segura River and is stored in a reservoir without prior treatment. This water source could facilitate the incorporation of aquatic microorganisms into the soil, favoring the presence of certain microbial communities adapted to these conditions, as described in previous studies [41,44]. The phyla Planctomycetota, Actinobacteriota, Bacteroidota, Chloroflexi, Myxococcota, and Firmicutes play a key role in the synthesis and transformation of dissolved organic matter in soil, regulating its composition and distribution [45]. In particular, the richness and diversity of Planctomycetota has been associated with the history of soil and its organic matter content, as well as with the availability of substrates of plant origin and the structure of the microbial ecosystem [46].

The general reduction of bacterial communities between Time 0 and Time 1 may be linked to the decrease in the area of arable soil as a result of agricultural preparation work, as well as to the reduction in soil organic matter content after the removal of plant remains from the previous crop. This could have induced a decrease in the availability of organic nitrogen and an increase in soil pH [37], unfavorable conditions for the proliferation of Actinobacteriota [38]. This phenomenon explains the significant and progressive reduction of this phylum during the study period. As for Bacteroidota, its decrease in relative abundance between Time 0 and Time 1 is associated with a reduction in the availability of polysaccharides of plant origin, which has been reported in studies on the degradation of organic matter in agricultural soils [36]. Furthermore, the widespread decrease in soil organic matter could have negatively impacted the presence of this phylum [47]. Additional studies have reported that both Actinobacteriota and Acidobacteriota are sensitive to variations in soil salinity and pH, which could explain their decline throughout the study [48].

### 4.2. Dynamics of Soil Bacterial Communities with Anti-Weed Mesh (S0, S2-T1, S2-T2)

These results suggest that the S2-T1 group experienced a transition phase in bacterial composition, probably in response to the conditions imposed by the anti-weed mesh. The greater dispersion observed in S2-T1 could reflect an unstable community in the process of adaptation, while the similarity between S0 and S2-T2 would indicate a tendency towards structural recovery of the bacterial soil microbiome over time. This behavior coincides with previous studies in which it has been reported that the installation of plastic covers can induce temporary changes in the soil bacterial community before reaching a new ecological equilibrium [49]. Previous studies have shown that the use of biodegradable and non-biodegradable plastics in agricultural soils significantly alters beta diversity, affecting the composition of microbial communities and modifying the functionality of the soil ecosystem [50].

From a functional perspective, these changes could have implications for essential soil processes, such as organic matter decomposition and nutrient cycling. The use of plastic mulches has been reported to influence the carbon and nitrogen cycle in soil, altering the metabolic activity of certain key bacterial communities [51]. The recovery observed in S2-T2 suggests that the soil bacterial community has the capacity to adapt to the presence of the anti-weed mesh over time, which could influence the stability of the agricultural ecosystem in the long term [52].

From Time 0 to Time 1, the increase experienced by the Firmicutes phylum could be related to an increase in soil pH due to land preparation practices, since a high pH can solubilize organic matter, increase denitrification potential, and stimulate microorganisms from the Firmicutes phylum [47,53].

On the other hand, the Bacteroidota phylum experienced a significant decrease in S2-T1. This decrease could be due to the increase in pH and a possible excess of moisture caused by the low soil aeration under the plastic cover [54]. Most of the phyla in S2 showed a relative abundance evolution similar to the study block S1 (Figure S4, Supplementary Materials). However, the Acidobacteriota phylum in S2-T2 suffered a reduction in abundance that was not detected in S1-T2, where it maintained more constant levels. It is understood that factors such as decreased pH, constant humidity near the root bulb, and low soil aeration influence Acidobacteriota [55]. However, it should be noted that soil pH and electrical conductivity were monitored throughout the study in the saturated 1:2 extract (soil:water), but no substantially relevant changes were detected, and very similar data were obtained. Therefore, due to the complexity of the study, the local conditions, the time elapsed, and the size of the experimental plot, it cannot be assured that neither the pH nor the electrical conductivity experienced localized changes throughout the duration of the study.

Furthermore, the Proteobacteria phylum experienced a more pronounced reduction in S2-T1 than in S1-T1. Some studies indicate that Proteobacteria are favored in long-term fertilized agricultural soils [56]. It is presumed that conditions in S2, with the plastic cover, concentrate more nitrogen fertilizers in the root bulb by reducing evaporation compared to S1.

Phyla such as Methylomirabilota, Firmicutes, Patescibacteria, Nitrospirota, Others, Latescibacterota, Desulfobacterota, Bdellovibrionota, and Thermoplasmatota did not exceed 5% relative abundance in S0 and S2-T2, while in S2-T1, they reached just over 11%, partly due to the increase in Firmicutes.

### 4.3. Comparison of Metagenomic Evolution in Soils with and Without Anti-Weed Mesh

#### 4.3.1. Alpha Diversity: Combined Impact of Time with Anti-Weed Control Mesh and Without Anti-Weed Control Mesh from the Start

This comparison aims to analyze the joint metagenomic evolution of the soil from Time 0 to 18 months, considering both the soil without a plastic cover and the soil with a plastic cover.

**Figure 4:**
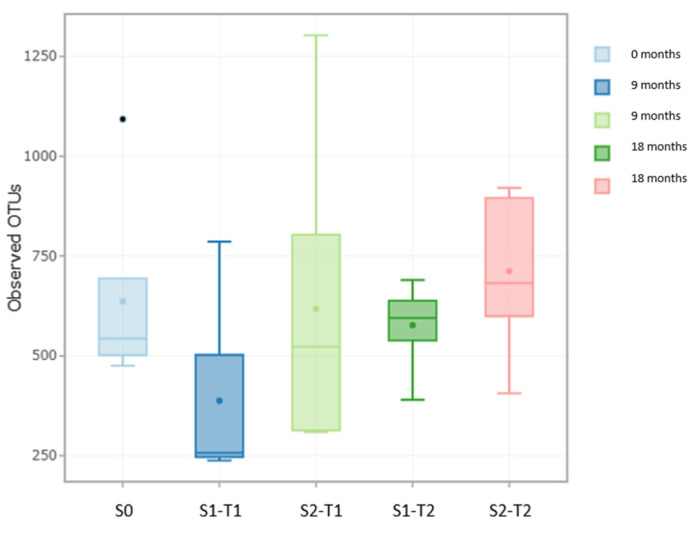
Representation of the intrasample alpha diversity of samples S0 for Time 0, S1 for Time 1 and Time 2 (*n* = 5), and S2 for Time 1 and Time 2 (all samples with *n* = 5). The point placed in each diagram indicates the arithmetic mean of the 5 samples that make up each study block.

The results presented above showed significant differences between samples S0 and S1-T1 in terms of OTU richness. Furthermore, significant differences were found in evenness between samples S0 and S2-T1 (Figure 4).

These results indicate that samples S1-T1 experienced a significant decrease in bacterial community richness, which is consistent with the findings reported in previous sections. In contrast, samples S2-T1 did not present a drastic reduction in the microbial population, but did show a decrease in community evenness [52,57].

Finally, no significant differences were found between samples S0 and S1-T2 or between S0 and S2-T2 in terms of either richness or evenness [58,59]. These results suggest that the most pronounced changes in the soil bacterial community occurred during the first 9 months of the study in both treatments (Figure 4).

#### 4.3.2. Beta Diversity: Structural Contrast Between Treatments and Times from the Start

**Figure 5:**
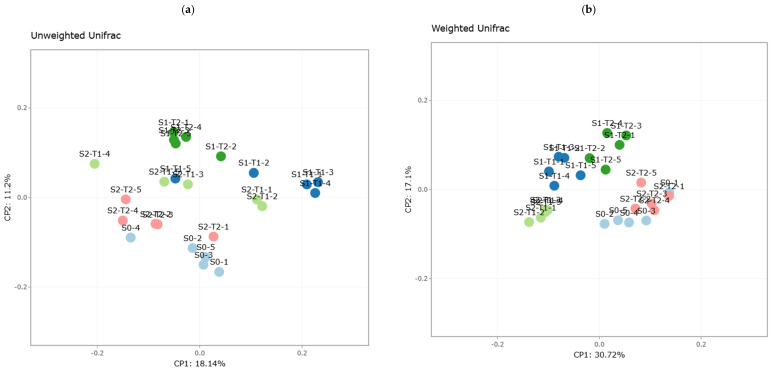

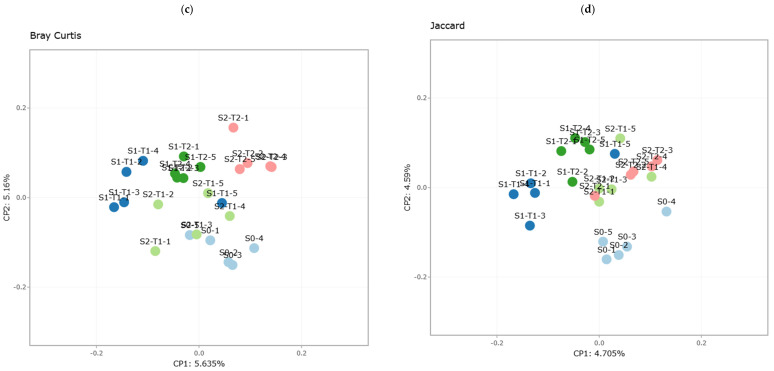
Principal coordinate analysis (PCoA) based on different beta diversity metrics for the sample groups. Each sample group is represented by a different color. (**a**) Unweighted Unifrac, (**b**) weighted Unifrac, (**c**) Bray-Curtis, and (**d**) Jaccard for the sample groups S0, S1-T1, S1-T2, S2-T1, and S2-T2.

In the unweighted Unifrac plot (Figure 5a), the five sample groups show a relatively tight clustering within each study block, indicating a general taxonomic closeness in terms of presence/absence of taxa. This suggests that although there are differences between treatments, most microbial groups share a common phylogenetic basis, albeit with minor variations in OTU composition.

On the other hand, in the weighted Unifrac plot (Figure 5b), a clearer separation between groups is observed, indicating differences in the relative abundance of taxa present in each experimental condition [60,61]. The more defined clustering suggests that changes in the abundance of certain microorganisms have been more decisive in differentiating treatments with and without an anti-weed mesh.

Bray–Curtis analysis (Figure 5c) reveals that groups S1-T1 and S1-T2 present the lowest abundance of phylotypes, reaffirming that the greatest reduction in bacterial richness occurred in the soil without weed control netting, particularly in the first 9 months of the study. This result is consistent with the hypothesis that soil preparation, together with exposure to environmental factors, causes a greater disruption in the soil bacterial community.

In contrast, the Jaccard-based PCoA analysis (Figure 5d) shows that groups S2-T1 and S2-T2 exhibit a greater presence of phylotypes, suggesting that weed control netting favors the retention of certain taxa in soil.

These results confirm that differences in beta diversity are influenced by both the presence or absence of weed control and the time elapsed since planting. The greater stability in weighted Unifrac and the greater retention of phylotypes in the weed control groups suggest that this type of agronomic management can modulate the dynamics of microbial communities in the long term, possibly by conserving soil moisture, reducing physical disturbance, and stabilizing the microbial niche.

#### 4.3.3. Taxonomic Profile: Changes in Distribution and Dominance of Key Taxa Across All Treatments

For the sample set S0, S1-T1, S1-T2, S2-T1, and S2-T2, a total of 37 phyla, 110 classes, 253 orders, 377 families, 640 genera, and 1267 species were identified in all soil samples (*n* = 25).

The bacterial dominant phyla in all soil samples, in decreasing order of relative abundance, were Planctomycetota, Proteobacteria, Actinobacteriota, Bacteroidota, Acidobacteriota, Chloroflexi, Gemmatimonadota, Verrucomicrobiota, Crenarchaeota, and Myxococcota, representing more than 90% of the readings performed, except in groups S1-T1 and S1-T2, where this percentage was approximately 88% (Table 2).

This difference between the S0 and S2 sample groups (Time 1 and Time 2) compared to the S1 group (time 1 and 2) was due to the Firmicutes phylum, which was the bacterial community that experienced the greatest change throughout the study [62]. In S0 (Time 0), its relative abundance was 0.92%, increasing significantly to 7.30% in S1-T1 (Time 1.9 months) and then decreasing to 5.96% in S1-T2 (Time 2, 18 months). Previous studies have shown that Firmicutes are sensitive to an increase in the organic matter content in soil, which would explain their progressive reduction as microbial activity stabilizes soil conditions [47,53].

The phyla Methylomirabilota, Firmicutes, Patescibacteria, Nitrospirota, Others, Latescibacterota, Desulfobacterota, Bdellovibrionota, and Thermoplasmatota did not exceed 6.5% of the total relative abundance in groups S0 and S2 (Time 1 and Time 2), while in S1 (time 1 and 2), they reached values higher than 11%, largely due to an increase in Firmicutes.

**Figure 6:**
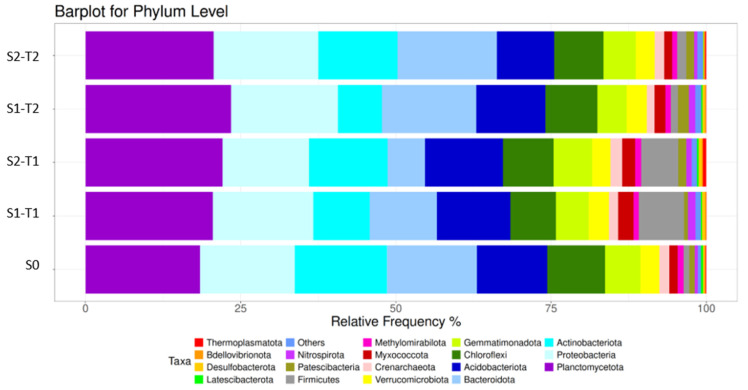
Relative abundance of bacterial phyla in soil without a weed control net (S1) and with a weed control net (S2) for Time 1, Time 2, and Time 0 (S0). Each bar indicates the relative proportion of each phylum as a function of time, showing the variations in soil microbial composition.

By observing the evolution of the relative abundance of taxa throughout the study (Figure 6), the effect of nitrogen fertilization on the microbial dynamics of the phyla Actinobacteriota and Acidobacteriota was identified in both group S1 (without an anti-weed mesh) and S2 (with an anti-weed mesh). In S1, Actinobacteriota showed a progressive and significant reduction in their relative abundance levels, while in S2, this decrease was more moderate in the first 9 months and stabilized in the final 18 months. In contrast, the Acidobacteriota phylum showed the opposite trend, increasing in S1 over time, but remaining more constant in S2. These results agree with previous studies suggesting that prolonged application of nitrogen fertilization can cause soil acidification, favoring the growth of Acidobacteriota and reducing the relative abundance of Actinobacteriota [56].

Regarding the evolution of the Bacteroidota phylum, it was observed that the reduction in its abundance was more pronounced in S2 compared to S1. This suggests that as the plant increased its water demand with the growth of the root system, the temporary accumulation of water under the anti-weed mesh progressively decreased, leading to a what could have caused a decrease in soil pH during the last 9 months of the study. This change favored the growth of Bacteroidota, which usually thrive in conditions of lower pH and high availability of dissolved organic matter [54].

The impact of plastic mulch was also reflected in phyla such as Nitrospirota, Latescibacterota, and Desulfobacterota, whose relative abundance decreased in group S2 after 18 months. The combination of localized fertigation with plant-based organic matter and moisture retention under the anti-weed mesh may have reduced soil pH and favored the accumulation of fertilizers in the soil’s wet bulb, creating less favorable conditions for these phyla. Previous research suggests that Nitrospirota thrive in soils with neutral pH and high nutrient levels, with a macro- and microaggregate structure that facilitates their establishment [63].

These findings highlight how the combination of fertilization and weed control can modify the microbial structure of soil, influencing its stability and the resilience of different taxa over time.

#### 4.3.4. Soil Bacterial Functionality: Predicting Metabolic Pathways Using KEGG

To identify the possible metabolic pathways active in the microbial communities detected, a potential taxonomic profile of the soil samples was performed and compared with the KEGG database. The potential metabolic pathways were estimated using computer software that predicts functional abundances based on marker gene sequences, but they should always be interpreted with caution. It should be taken into account that they are predictions and that in the study, they were not verified in the field by any other means.

Of the 3554 metabolic pathways detected, 2520 obtained significant results (*p*-value <0.05) for the combined effect of anti-weed mesh and time, according to the ANOVA using Fisher’s exact test.

**Figure 7:**
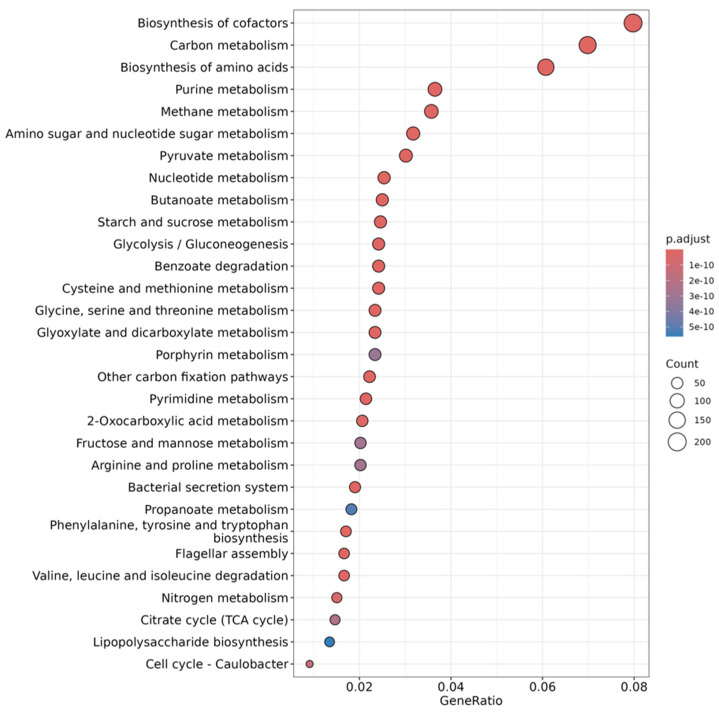
Overrepresented KEGG features (top 30 most significant pathways (with the lowest *p*-value)) for all treatments. Each dot is colored by its *p*-value, and its size is based on the number of features observed in the given enriched pathway.

From these results, 89 metabolic pathways were selected for their predominance in these samples, of which 54 pathways exceeded 1% relative abundance. These pathways are mainly related to cofactor biosynthesis, carbon metabolism, amino acid biosynthesis, purine metabolism, methane metabolism, and amino sugar and sugar-nucleotide metabolism [64,65] (Figure 7).

These metabolic processes are fundamental for the functionality of the soil microbiome and can be modulated by factors such as nutrient availability, moisture retention, and the structural composition of the microbial community [66]. The variation in the expression of these pathways over time suggests that agronomic management can influence the stability and dynamics of microbial metabolic functions in soil [67].

### 4.4. Comparison of Soil Metagenomic Dynamics with and Without Anti-Weed Mesh over a 9-Month Period

#### 4.4.1. Alpha Diversity: Combined Impact of Time and Anti-Weed Control Mesh and Whithout Anti-Weed Control Mesh 

**Figure 8:**
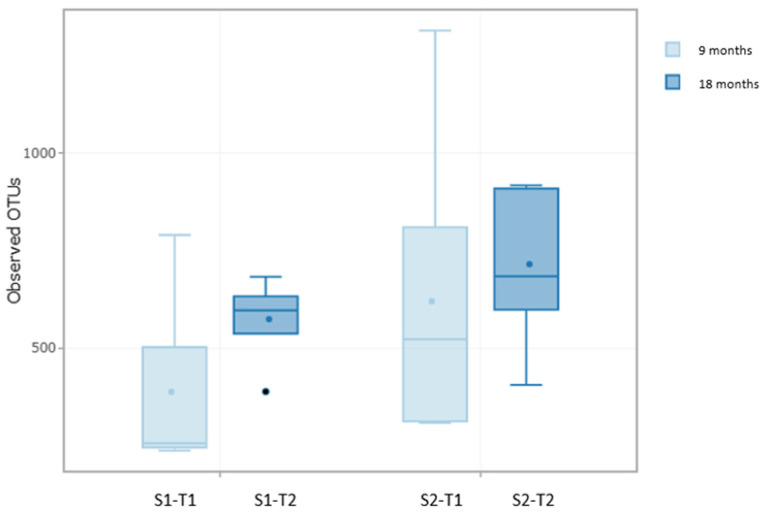
Representation of the intrasample alpha diversity of samples S1 and S2 for both Time 1 (*n* = 5) and Time 2 (*n* = 5). The point placed in each diagram indicates the arithmetic mean of the 5 samples that make up the study block.

The metagenomic evolution of the soil was analyzed during the last 9 months of the study, comparing the conditions without anti-weed netting (S1-T1 and S1-T2) and with anti-weed netting (S2-T1 and S2-T2). Alpha and beta diversity, taxonomic profile, and metabolic pathways were evaluated, with the aim of determining the impact of time and the use of netting on the microbial composition of soil. The results indicated that there were no significant differences in the richness of OTUs (Figure 8). Similarly, community evenness did not differ significantly between groups, showing similar values. However, when comparing the time variable in samples with anti-weed netting (S2-T1 and S2-T2), a significant improvement in evenness was detected (*p* = 0.00885), suggesting that the netting contributed to stabilizing the microbial composition over time [29,35].

#### 4.4.2. Beta Diversity: Structural Contrast Between Treatments with and Without Anti-Weed Mesh and Times

**Figure 9:**
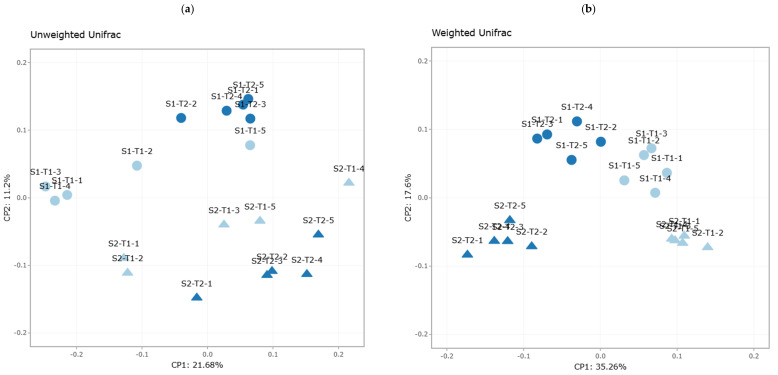

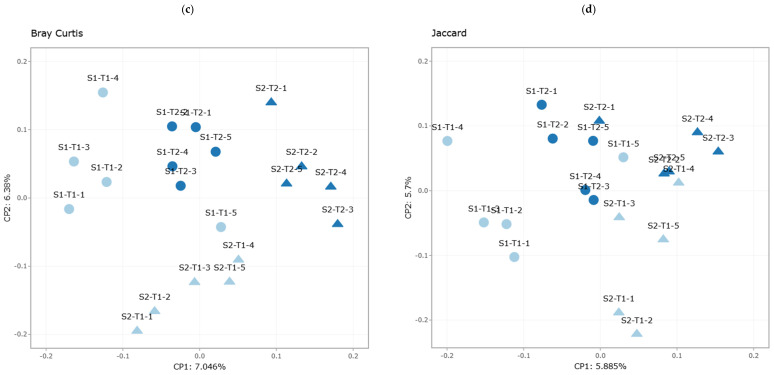
Principal coordinate analysis (PCoA) based on different beta diversity metrics for the sample groups. Each sample group is represented by a different color. (**a**) Unweighted Unifrac, (**b**) weighted Unifrac, (**c**) Bray-Curtis, and (**d**) Jaccard for the sample groups S1-T1, S1-T2, S2-T1, and S2-T2.

Beta diversity analysis using unweighted and weighted Unifrac revealed a clear grouping of the samples according to their treatment and sampling time. The weighted Unifrac plot showed a defined clustering, indicating similarities in the presence and relative abundance of the taxa in each group (Figure 9b). In contrast, the unweighted Unifrac showed a clear separation between treatments, with samples from groups S2-T1 and S2-T2 located at the bottom of the coordinate space, reflecting greater taxonomic stability in the soil with an anti-weed mesh (Figure 9a).

The Bray–Curtis and Jaccard indices also confirmed differences in bacterial community composition, although with some dispersion in the samples (Figure 9c,d). In general, soils with an anti-weed mesh showed less structural variability, suggesting a stabilizing effect on the soil bacterial community [56].

#### 4.4.3. Taxonomic Profile: Changes in Distribution and Dominance of Key Taxa with and Without Anti-Weed Mesh

For samples S1-T1, S1-T2, S2-T1, and S2-T2, a total of 37 phyla, 107 classes, 245 orders, 362 families, 597 genera, and 1158 species were identified in all soil samples (*n* = 20).

**Figure 10:**
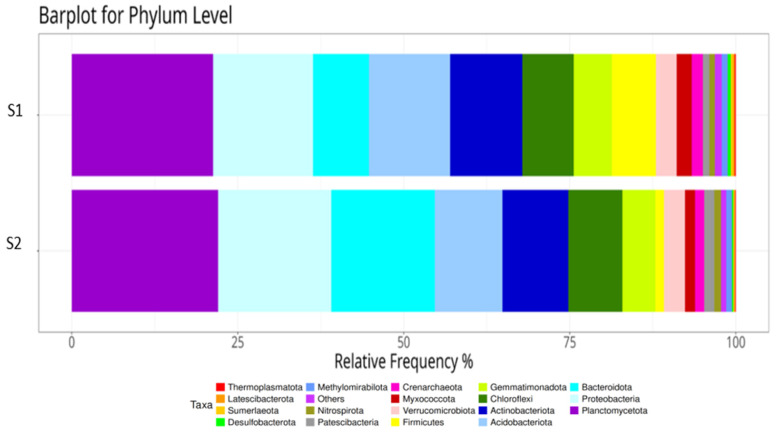
Mean relative abundance of bacterial phyla in soil without weed netting (S1) and with weed netting (S2). Each bar indicates the relative proportion of each phylum as a function of time, showing variations in soil microbial composition.

The bacterial dominant phyla in all soil samples were (ordered from highest to lowest percentage of relative abundance) Planctomycetota, Proteobacteria, Acidobacteriota, Actinobacteriota, Bacteroidota, Chloroflexi, Firmicutes, Gemmatimonadota, Verrucomicrobiota, and Myxococcota, representing more than 93% (Figure 10). The Firmicutes phylum is the bacterial community with the greatest difference in this comparison. In sample group S1, a presence of 6.63% was detected compared to 1.29% in sample group S2 (both groups for times 1 and 2). The phyla Crenarchaeota, Patescibacteria, Nitrospirota, Others, Methylomirabilota, Desulfobacterota, Sumerlaeota, Latescibacterota, and Thermoplasmatota did not exceed 6.5% of the total relative abundance in either group.

This comparison evaluates the relative abundances of the bacterial phyla between blocks S1 and S2, the influence that the anti-weed net had on the growth of the bacterial phyla, the elapsed time, and the time–anti-weed net interaction. Visually, we can see in Table 3 how the vast majority of bacterial phyla were significantly influenced by the anti-weed net, by the elapsed time, or by the interaction of both. The four dominant phyla in both study blocks—Planctomycetota, Proteobacteria, Acidobacteriota, and Actinobacteriota—were influenced by the weed control mesh, time, or the interaction of both (Figure 10). These results coincide with several similar studies carried out to date, but under different conditions [68].

It is important to highlight that the 2 phyla with the highest relative abundance of the 12 represented in Table 3, Planctomycetota and Proteobacteria, had a relatively higher abundance in group S2 than in group S1, implying that the culture conditions applied in the aforementioned group favor their growth and development compared to S1. However, when considering all the phyla together, 7 of the 12 represented were detected with greater frequency and intensity in group S1. The results obtained for the Bacteroidota and Firmicutes phyla are striking, as these two phyla show totally contrary results, clearly indicating under which culture conditions they prefer to develop. Possibly, the same is true for the Gemmatimonadota, Myxococcota, and Nitrospirota phyla, which were significantly influenced by the time and mesh variables.

#### 4.4.4. Functional Prediction of Soil Bacterial Community Using KEGG: Analysis of Metabolic Pathways and Their Ecological Implication

The taxonomic profile of the samples was compared with the KEGG database to assess the predominant metabolic pathways in each condition. Of the 1654 metabolic pathways detected, 1366 showed significant differences depending on treatment and time (*p* < 0.05, Fisher’s exact test).

**Figure 11:**
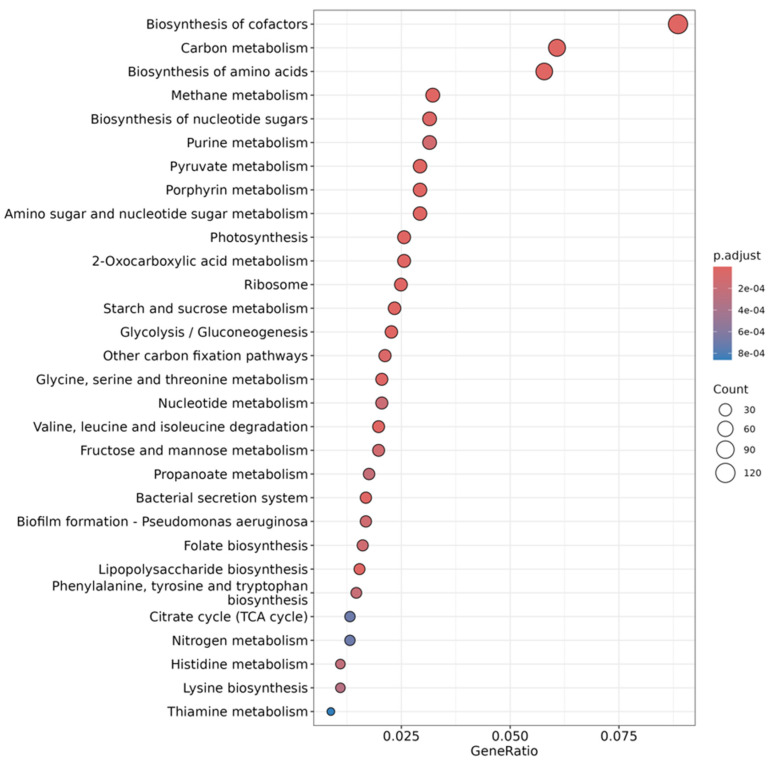
Overrepresented KEGG features (top 30 most significant pathways (with the lowest *p*-value)) for mesh and without mesh treatments. Each dot is colored by its *p*-value, and its size is based on the number of features observed in the given enriched pathway.

Of these, 66 pathways were selected for their relevance in microbial metabolism, of which 53 exceeded 1% relative abundance. These pathways are mainly related to cofactor biosynthesis, carbon metabolism, amino acid biosynthesis, methane metabolism, and sugar-nucleotide biosynthesis (Figure 11), suggesting that agronomic management influences soil microbial functionality. The regulation of these processes is key to the stability of agricultural ecosystems and their ability to recover after disturbances [56].

## 5. Conclusions

The results obtained in this study confirm that mechanical soil preparation operations before planting exert a significant impact on the population dynamics of the soil bacterial community. The disruption of the soil structure caused by the fragmentation of macro- and microaggregates exposes a larger soil surface to climatic conditions, limiting the stability of microbial niches during the first months after planting.

The effect of this perturbation was more pronounced in the group without an anti-weed mesh (S1), where the reduction in OTU richness was considerably higher compared to the group with an anti-weed mesh (S2). During the first 9 months of the study, S1 showed a decrease of more than 229 OTUs compared to S2, indicating that soil covered with an anti-weed mesh contributes to cushioning the negative effects of mechanical work on the soil bacterial community. In addition, at 18 months, S2 maintained a higher microbial richness, with 135 additional OTUs compared to S1, suggesting that the microenvironmental conditions generated by the anti-weed mesh favor the establishment and development of more stable and diverse microbial communities.

From a taxonomic point of view, the comparative analysis between groups S0-S1 and S0-S2 revealed greater diversity in S2, with the detection of 2 phyla, 3 classes, 17 orders, 27 families, 53 genera, and 117 additional species compared to S1. This increase in diversity suggests that soil cover not only protects the existing microbial community, but also favors the colonization of new taxa, suggesting that weed control netting does not reduce its population as drastically and remains constant after the last 9 months of the study. Soil covered with a weed control mesh not only attenuates the initial impact of mechanical work but also favors greater bacterial diversity and stability over time. These results have important implications for sustainable soil management in semi-arid agroecosystems, suggesting that the use of plastic mulches may be an effective strategy to conserve bacterial biodiversity and optimize soil health in perennial crops such as sweet orange.

Finally, it should be emphasized that this study focused solely on the evolution of bacterial population dynamics.

## Figures and Tables

**Table 1:** Characteristics and nomenclature of the soil samples used in the study.

Sample	Time Elapsed Since Planting (Months)	Anti-Weed Mesh
S0	0	No
S1-T1	9	No
S2-T1	9	Yes
S1-T2	18	No
S2-T2	18	Yes

**Table 2:** Relative abundance expressed as a percentage of bacterial taxa in the sample groups S0, S1-T1, S1-T2, S2-T1, and S2-T2. The last column shows the *p*-value calculated from the ANOVA for the time variable.

Taxa	S0	S1-T1 (Without Mesh)	S1-T2 (Without Mesh)	S2-T1 (With Mesh)	s2-T2 (With Mesh)	Time (*p*-Value)
Planctomycetota	18.47	20.50	23.45	22.06	20.64	0.00
Proteobacteria	15.20	16.18	17.20	13.93	16.85	0.02
Actinobacteriota	14.90	9.09	7.11	12.64	12.75	0.00
Bacteroidota	14.46	10.80	15.19	6.03	16.00	0.00
Acidobacteriota	11.31	11.84	11.14	12.58	9.23	0.00
Chloroflexi	9.33	7.35	8.36	8.14	7.92	0.00
Gemmatimonadota	5.69	5.26	4.69	6.20	5.22	0.00
Verrucomicrobiota	3.05	3.29	3.28	2.96	3.03	0.92
Crenarchaeota	1.59	1.44	1.22	1.88	1.52	0.34
Myxococcota	1.37	2.48	1.75	2.06	1.27	0.00
Methylomirabilota	0.93	0.83	0.87	0.97	0.83	0.76
Firmicutes	0.92	7.30	1.14	5.96	1.43	0.00
Patescibacteria	0.87	0.63	1.76	1.32	1.26	0.00
Nitrospirota	0.51	1.21	1.03	0.85	0.52	0.00
Others	0.33	0.38	0.66	0.84	0.91	0.00
Latescibacterota	0.35	0.20	0.22	0.32	0.08	0.03
Desulfobacterota	0.23	0.47	0.33	0.47	0.11	0.00
Bdellovibrionota	0.14	0.21	0.20	0.15	0.13	0.00
Thermoplasmatota	0.12	0.02	0.04	0.56	0.21	0.00

**Table 3:** Mean relative abundance expressed as a percentage of bacterial taxa in the sample groups S1-T1, S1-T2, S2-T1, and S2-T2. The last 3 columns show the *p*-values calculated for the ANOVA for the variables mesh, time, and the mesh–time interaction.

Taxa	S1 (Without Mesh)	S2 (With Mesh)	Mesh (*p*-Value)	Time (*p*-Value)	Time-Mesh (*p*-Value)
Planctomycetota	21.28	22.04	0.28	0.26	0.00
Proteobacteria	15.06	17.02	0.03	0.00	0.09
Acidobacteriota	12.21	10.18	0.33	0.00	0.03
Actinobacteriota	10.87	9.93	0.00	0.12	0.09
Bacteroidota	8.41	15.59	0.00	0.00	0.00
Chloroflexi	7.74	8.14	0.52	0.12	0.02
Firmicutes	6.63	1.29	0.19	0.00	0.02
Gemmatimonadota	5.73	4.96	0.00	0.00	0.58
Verrucomicrobiota	3.12	3.15	0.38	0.92	0.91
Myxococcota	2.27	1.51	0.00	0.00	0.30
Crenarchaeota	1.66	1.37	0.12	0.25	0.85
Patescibacteria	0.97	1.51	0.60	0.00	0.00
Nitrospirota	1.03	0.77	0.00	0.01	0.20
Others	0.86	0.89	0.00	0.00	0.00
Methylomirabilota	0.90	0.85	0.58	0.52	0.34
Desulfobacterota	0.47	0.22	0.04	0.00	0.00
Sumerlaeota	0.16	0.21	0.00	0.00	0.00
Latescibacterota	0.26	0.15	0.00	0.00	0.00
Thermoplasmatota	0.29	0.13	0.00	0.00	0.00

## Data Availability

The data presented in this study are available in the present work.

## Supplementary Materials

The following supporting information can be downloaded at https://www.mdpi.com/article/10.3390/plants14121781/s1. Figure S1: Representation of the alpha diversity of samples S0 for time 0, S1 for time 1 (9 months) and time 2 (18 months). Mean values of the samples (*n* = 5). The central point of each diagram indicates the arithmetic mean of the five samples that make up each study block; Table S1: Relative abundance expressed as a percentage of bacterial taxa in the sample groups S0, S1-T1 and S1-T2, and the *p*-value calculated from the Anova for the time variable; Figure S2: Relative abundance of bacterial phyla in soil without weed control mesh (S1) throughout the study, at Time 0 (S0), S1-T1 (Time 1, 9 months) and S1-T2 (Time 2, 18 months). Each bar indicates the relative proportion of each phylum as a function of time, showing variations in soil microbial composition. Figure S3: Representation of the alpha diversity of samples S0 for time 0, S2 for time 1 (9 months) and time 2 (18 months). Mean values of the samples (*n* = 5). The central point of each diagram indicates the arithmetic mean of the five samples that make up each study block. Table S2: Relative abundance expressed as a percentage of bacterial taxa in sample groups S0 and S2 (time 1 and 2). The last column shows the *p*-value calculated from the Anova for the time variable. Figure S4: Relative abundance of bacterial phyla in soil with weed control mesh (S2) throughout the study, at Time 0 (S0), S2-T1 (Time 1, 9 months) and S2-T2 (Time 2, 18 months). Each bar indicates the relative proportion of each phylum as a function of time, showing variations in soil microbial composition.
