# Supplementary material for: Evolution of the Soil Bacterial Community as a Function of Crop Management: A Metagenomic Study in Orange Tree (Citrus sinensis) Plantations
Source: Plants (Basel). 2025 Jun 11;14(12):1781. doi: 10.3390/plants14121781 (PMC12196881; doi:10.3390/plants14121781)
Supplement: Supplementary file 1 [file plants-14-01781-s001.zip › plants-3615491-supplementary.pdf]

Plants-3615491

EVOLUTION OF SOIL BACTERIAL COMMUNITY AS A FUNCTION OF CROP MANAGEMENT: A METAGENOMIC STUDY IN *Citrus sinensis* PLANTATIONS

SUPPLEMENTARY MATERIAL

1. Extended description of experimental agricultural plot

The experimental plot, with an area of 7,500 m^2, has contained, since March 2023, a plantation of sweet orange (*Citrus sinensis* L. Osbeck), variety 'Navelina' on *Citrus macrophylla* Wester rootstocks. Previously, and for approximately 25 years, the plot included a plantation of lemon (*Citrus limon* L. Osbeck), variety 'Fino' on bitter orange (*Citrus aurantium* L.) rootstocks. At the end of 2022, the lemon plantation was uprooted, after which the plot was subjected to excavation work, shredding, crushing of soil aggregates and levelling to ground level of the entire plot before planting the orange trees, using large specialized agricultural machinery. Orange cultivation was chosen for its representativeness in the southeastern area of Spain, where the plot is located, since citrus cultivation represents a significant proportion of the local agricultural sector.

The crop was established with a rectangular planting frame of 6 x 4 m and is managed under a drip fertigation system with four drippers per tree (2 L/h each). Irrigation is adjusted according to the water needs of the crop, monitored by a precision agricultural probe system, which allows continuous control of soil moisture and ensures optimization of water use. During the study, climatic conditions were kept homogeneous for all study blocks.

2. Figures and tables

Figure S1. Representation of the alpha diversity of samples S0 for time 0, S1 for time 1 (9 months) and time 2 (18 months). Mean values of the samples (n=5). The central point of each diagram indicates the arithmetic mean of the five samples that make up each study block.

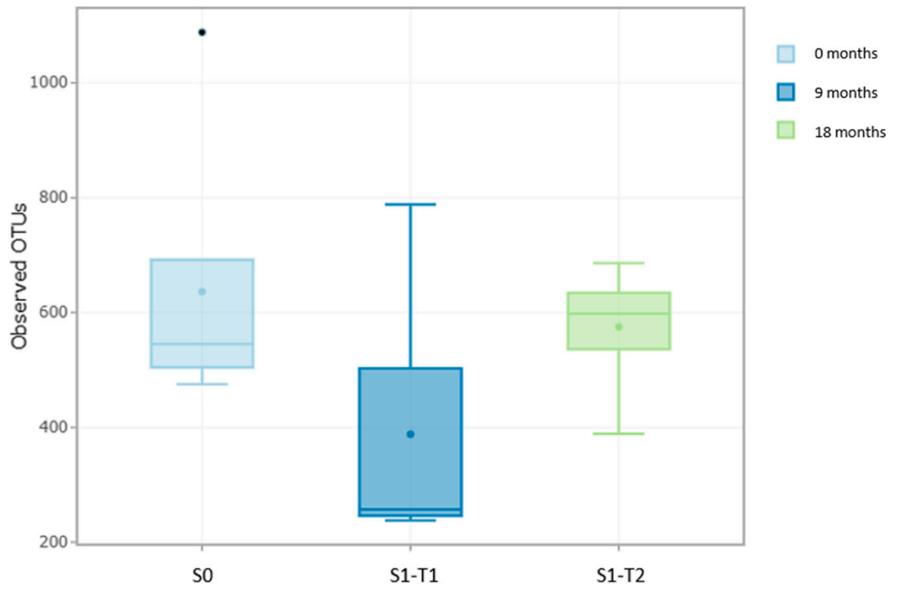

Table S1. Relative abundance expressed as a percentage of bacterial taxa in the sample groups S0, S1-T1 and S1-T2, and the p-value calculated from the Anova for the time variable.

Taxa	S0	S1-T1	S1-T2	Time (p-value)
Planctomycetota	18,47	20,50	23,45	0,00
Proteobacteria	15,20	16,18	17,20	0,31
Actinobacteriota	14,90	9,09	7,11	0,00
Bacteroidota	14,46	10,80	15,19	0,00
Acidobacteriota	11,31	11,84	11,14	0,79
Chloroflexi	9,33	7,35	8,36	0,00
Gemmatimonadota	5,69	5,26	4,69	0,08
Verrucomicrobiota	3,05	3,29	3,28	0,80
Crenarchaeota	1,59	1,44	1,22	0,58
Myxococcota	1,37	2,48	1,75	0,00
Methylomirabilota	0,93	0,83	0,87	0,75
Firmicutes	0,92	7,30	1,14	0,00
Patescibacteria	0,87	0,63	1,76	0,00
Nitrospirota	0,51	1,21	1,03	0,00
Others	0,33	0,38	0,66	0,00
Latescibacterota	0,35	0,20	0,22	0,00
Desulfobacterota	0,23	0,47	0,33	0,00
Bdellovibrionota	0,14	0,21	0,20	0,00
Sumerlaeota	0,11	0,26	0,21	0,00

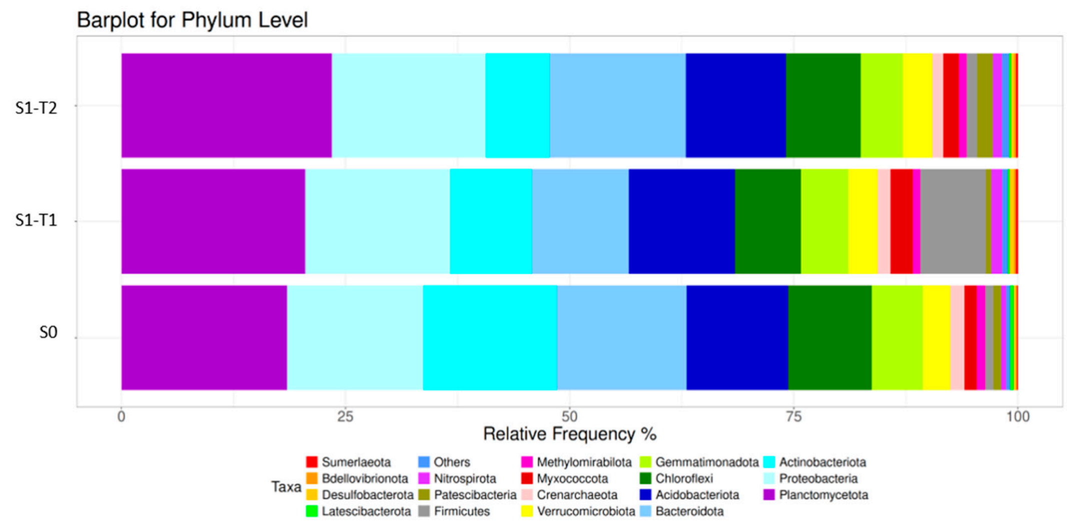

Figure S2. Relative abundance of bacterial phyla in soil without weed control mesh (S1) throughout the study, at Time 0 (S0), S1-T1 (Time 1, 9 months) and S1-T2 (Time 2, 18 months). Each bar indicates the relative proportion of each phylum as a function of time, showing variations in soil microbial composition.

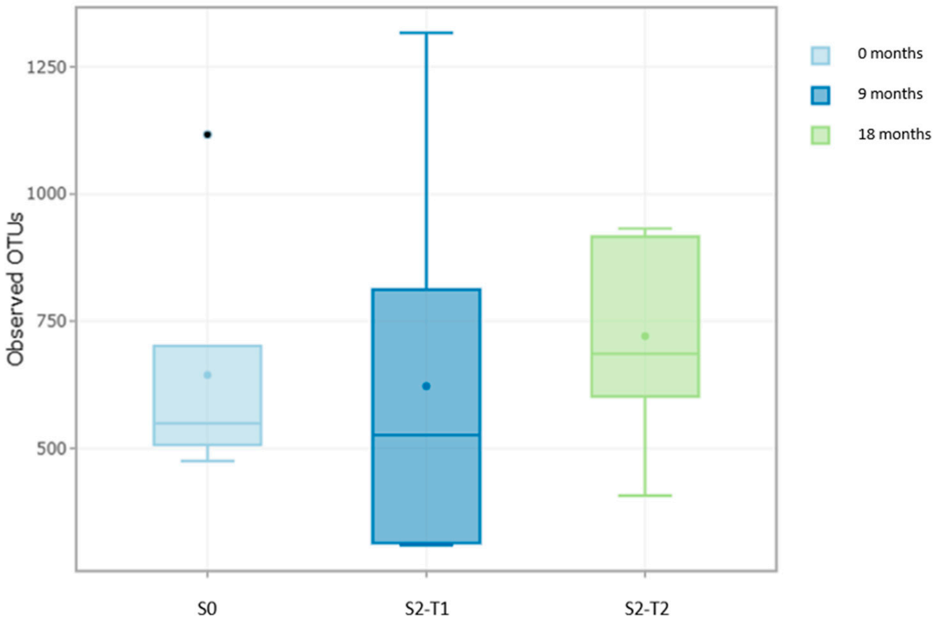

Figure S3. Representation of the alpha diversity of samples S0 for time 0, S2 for time 1 (9 months) and time 2 (18 months). Mean values of the samples (n=5). The central point of each diagram indicates the arithmetic mean of the five samples that make up each study block.

Table S2. Relative abundance expressed as a percentage of bacterial taxa in sample groups S0 and S2 (time 1 and 2). The last column shows the p-value calculated from the Anova for the time variable.

Taxa	S0	S2-T1	S2-T2	Time (p-value)
Planctomycetota	18,47	22,06	20,64	0,00
Proteobacteria	15,20	13,93	16,85	0,04
Actinobacteriota	14,90	12,64	12,75	0,02
Bacteroidota	14,46	6,03	16,00	0,00
Acidobacteriota	11,31	12,58	9,23	0,00
Chloroflexi	9,33	8,14	7,92	0,02
Gemmatimonadota	5,69	6,20	5,22	0,07
Verrucomicrobiota	3,05	2,96	3,03	0,98
Crenarchaeota	1,59	1,88	1,52	0,18
Myxococcota	1,37	2,06	1,27	0,00
Methylomirabilota	0,93	0,97	0,83	0,52
Firmicutes	0,92	5,96	1,43	0,00
Patescibacteria	0,87	1,32	1,26	0,12
Nitrospirota	0,51	0,852	0,52	0,00
Others	0,33	0,846	0,91	0,00
Latescibacterota	0,35	0,32	0,08	0,00
Desulfobacterota	0,23	0,47	0,11	0,00
Bdellovibrionota	0,14	0,15	0,13	0,00
Thermoplasmatota	0,12	0,56	0,21	0,00

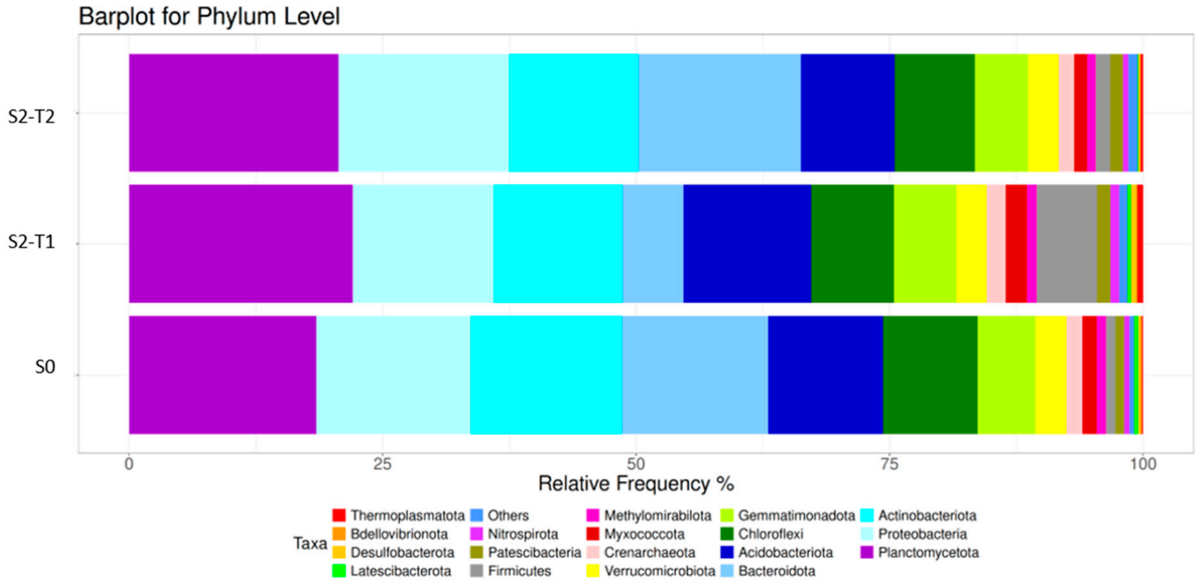

Figure S4. Relative abundance of bacterial phyla in soil with weed control mesh (S2) throughout the study, at Time 0 (S0), S2-T1 (Time 1, 9 months) and S2-T2 (Time 2, 18 months). Each bar indicates the relative proportion of each phylum as a function of time, showing variations in soil microbial composition.
